# Filtered Kombucha tea ameliorates the leaky gut syndrome in young and old mice model of colitis

**DOI:** 10.22038/ijbms.2019.36189.8622

**Published:** 2019-10

**Authors:** Nafiseh Pakravan, Fatemeh Kermanian, Elaheh Mahmoudi

**Affiliations:** 1 Division of Immunology, School of Medicine, Alborz University of Medical Sciences, Karaj, Iran; 2 Department of Anatomy, School of Medicine, Alborz University of Medical Sciences, Karaj, Iran; 3 Department of Mycology, School of Medicine, Alborz University of Medical Sciences, Karaj, Iran

**Keywords:** Age, Colitis, Kombucha tea, Leaky gut, ZO-1, ZO-2

## Abstract

**Objective(s):**

Zonula occludens proteins (ZO-1 and ZO-2) are important intracellular tight junction (TJ)-associated proteins that link the cell cytoskeleton to the trans-membrane TJ proteins. Destruction of TJ proteins is called the “leaky gut syndrome” and has been observed in some of the gastrointestinal diseases such as the inflammatory bowel disease (IBD). So, therapeutic approaches aim to restore the expression of TJ proteins and reduce intestinal permeability. Healing effect of Kombucha tea (KT), so-called long-life mushroom, on the gastrointestinal system, particularly its extraordinary healing effects on intestinal ulcers has been purported traditionally and rarely reported scientifically. This study aimed to investigate the therapeutic effect of filtered KT (fKT) in young and old mice model of colitis.

**Materials and Methods:**

Leaky gut was induced in two groups of young and old age using dextran sodium sulfate in drinking water for seven days. Then, fKT was administered to the mice affected by colitis and compared with the age-matched normal and untreated animals with colitis.

**Results:**

Survival rate of the fKT-treated young and old animals with colitis increased and weight loss decreased. Accordingly, digestive disorders characterized by bleeding and diarrhea were improved in fKT-treated mice. Molecular and histological examination indicated that expression of ZO-1 and ZO-2 was significantly improved in fKT-treated mice.

**Conclusion:**

Our results suggest KT as a promising therapeutic candidate to reduce intestinal permeability. Young animals with colitis showed more severe clinical signs and less survival rate than old mice with colitis, but this group responded better to fKT treatment than the old mice.

## Introduction

The intestinal barrier contains a layer of intestinal epithelium, which separates luminal contents from the interstitium tissue. The intestinal epithelium expresses integral trans-membrane proteins including occludin, claudins, junctional adhesion molecule (JAM), and tricellulin (1-4), making the complexes of tight junction (TJ) proteins. The intracellular domains of these intercellular transmembrane proteins interact with the cytosolic proteins, including Zonula Occludens (ZO) proteins (ZO-1 and ZO-2). Domains of ZO proteins are, in turn, connected to the cell cytoskeleton (1). The chain connection made from TJ protein domains to the actin cytoskeleton plays a critical role in regulation of TJ integrity and the signal transduction pathways (5).TJ permeability has been shown to be under the influence of a variety of factors such as food (6), hormones (7, 8), microbiota (9), and cytokines (10, 11). The destruction of any part of TJ building leads to passage of large particles of food, bacterial toxins, and other microorganisms into the bloodstream. This phenomenon is called the “leaky gut syndrome” or the “intestinal leakage syndrome” (12). Conditions such as infection, certain drugs such as antibiotics or non-steroidal anti-inflammatory drugs, long-term emotional stress, high levels of alcohol and sugar, and allergic foods can break down tight connections and play an important role in the development of intestinal leakage (13). In addition, several studies have documented that the age factor can affect the intestinal barrier structure and function (14-16). Increased intestinal permeability can predict the incidence and severity of gastrointestinal diseases, including IBS and IBD (16-18). So, reversing and reducing intestinal permeability is the goal of developing therapeutic approaches. However, none of the proposed approaches for restoration of the barrier have been so far proven effective (19). In addition, the available synthetic anti-colitis drugs are expensive and show side effects. Exploration of herbal drugs might provide suitable alternative anti-colitis formulations (20). One of the natural remedies is Kombucha tea (KT), a popular traditional fermented drink throughout the world (21-22). Healing effects of KT, on the gastrointestinal system, particularly its extraordinary effect on the healing of intestinal ulcers has been purported traditionally and reported scientifically (20-22). Also, we previously demonstrated that KT had rejuvenating effects on the skin of old mice (23). It prompted us to investigate if KT can also have therapeutic or relieving effects on the mice model of colitis. Therefore, KT was administered to young and old mice model of colitis and compared with the normal and age-matched untreated animals with colitis. Since age is an important factor affecting intestinal TJ structure and function, young and old mice were both included in the study.

## Materials and Methods

***Kombucha Tea (KT) preparation***

Black tea (Golestan, Tehran, Iran) was added to boiling water (1.2%w/v), mixed, and left to brew for 10 min. The tea was then filtered through a sterile sieve, and sucrose (10%) was dissolved in the tea. To prepare KT, 200 ml of the cooled black tea was inoculated with 3%w/v tea fungus plus 10%v/v KT liquid that was previously fermented. The collection was then left to ferment by incubating at 28 ^°^C for 14 days. To make filtered KT (fKT), the resultant fermented tea was centrifuged at 5000 rpm for 20 min and filtered using a 0.45 µm cellulose filter equipped with a vacuum pump (24). 

***Experimental groups and study design***

Male NMR mice were purchased from the Pasteur Institute Experimental Animal Center, Tehran, Iran. All animals were housed for one week before the experiments began in light- and temperature-regulated rooms at the conventional animal department of Alborz University of Medical Sciences. The animals were provided with food and water *ad libitum*. All experiments were approved by the local ethical committee (reference No Abzums.Rec.1395.51) and performed according to Animal Care and Use Protocol of Alborz University of Medical Sciences. 

**Figure 1:**
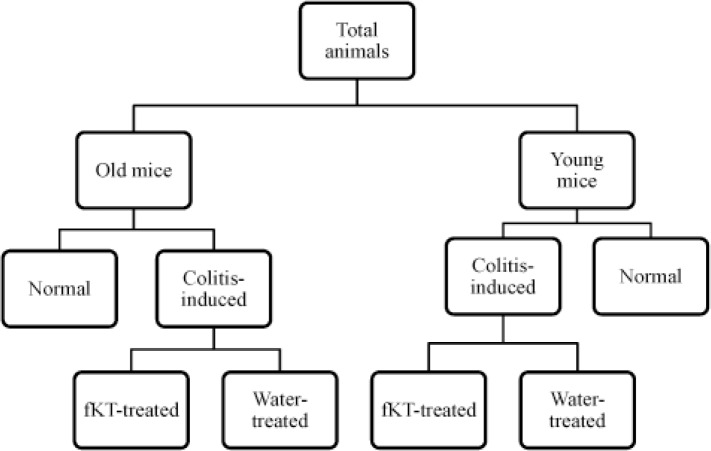
Experimental groups of the study

**Figure 2:**
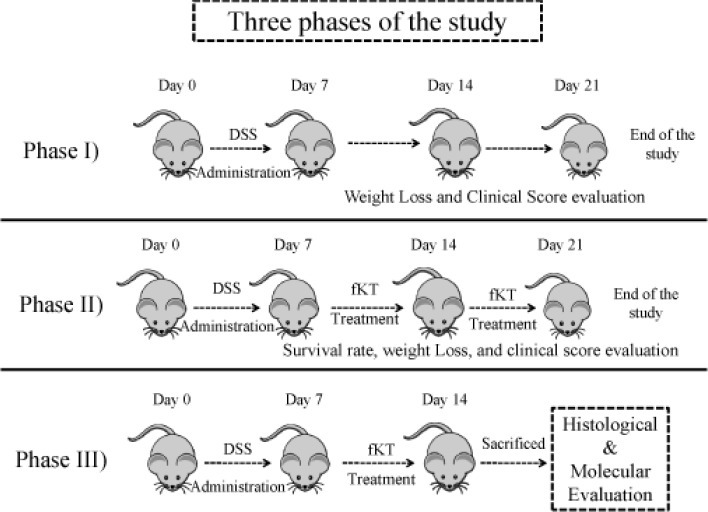
Three phases of the study through which experimental groups were designed and treated

**Figure 3:**
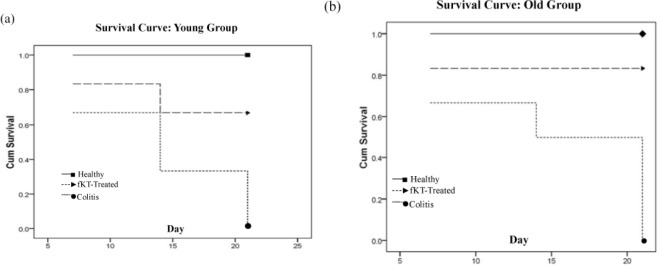
Survival curve of young (a) and old (b) groups including young and old healthy control, young and old DSS-induced colitis, and young and old DSS-induced colitis treated with fKT. Animals were monitored every day for the duration of the experiment. Survival rate was markedly increased by fKT treatment. Analysis made by Kaplan–Meier and compared using the log-rank test

**Figure 4:**
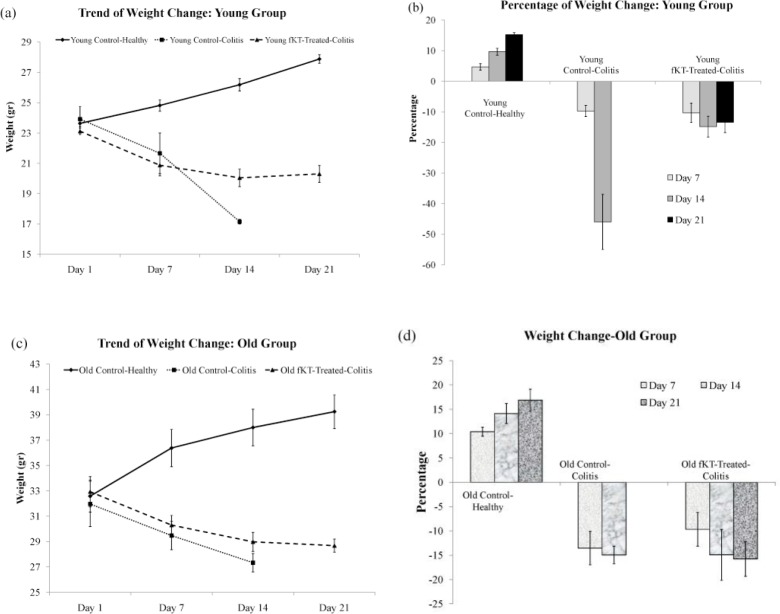
Trend of weight change and percentage of weight loss in the young (a, b) and old (c, d) groups including young and old healthy control, young and old DSS-induced colitis, and young and old DSS-induced colitis treated with fKT. Weight change and weight loss are remarkably affected by fKT treatment

**Figure 5:**
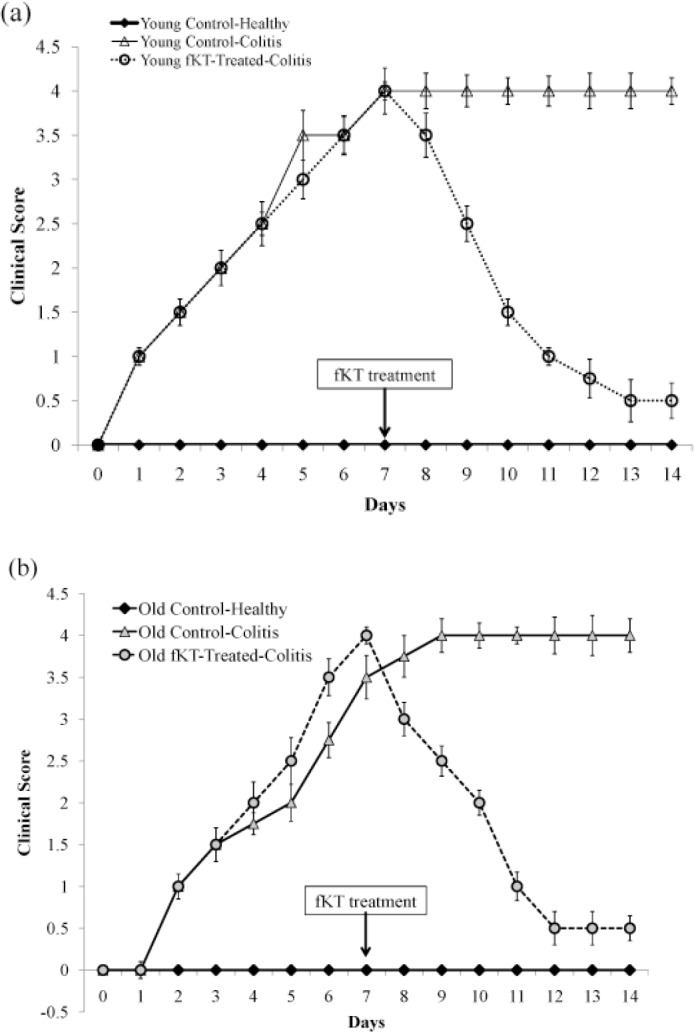
Clinical Score of the young (a) and old (b) DSS-induced colitis mice treated with fKT comparing with the age-matched healthy or DSS-induced colitis mice measured daily from the day of disease induction. In line with the survival rate and weight loss, the clinical score was also improved by fKT treatment. Data are presented as mean±SD

**Figure 6:**
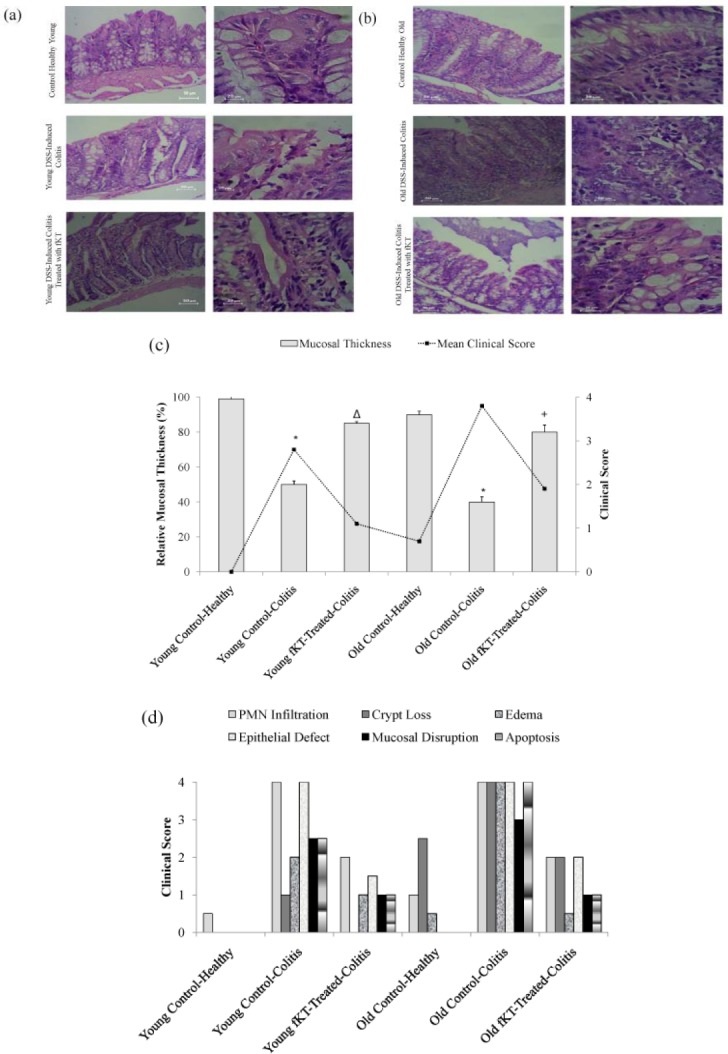
(a) H&E stained colon sections of healthy young animals, young DSS-induced colitis, and young DSS-induced colitis treated with fKT, (b) healthy old animals, old DSS-induced colitis, and old DSS-induced colitis treated with fKT comparing PMNs, cryptic loss, epithelial defect, mucosal disruption, apoptosis, edema, and mucosal thickness. Colitis was induced by administration of 3.5% DSS in drinking water for seven days, and treatment with fKT was performed for the next seven days. The picture was taken using an Olympus D330 digital camera (Olympus, Tokyo, Japan). The right panel with a magnification of x10 and the left with ×40. (c) Thickness changes in the colon of healthy young animals, young DSS-induced colitis, and young DSS-induced colitis treated with fKT, healthy old animals, old DSS-induced colitis, and old DSS-induced colitis treated with fKT. The thickness of mucosa colon was estimated relative to age-matched healthy animals in a total of six areas per sample with fixed interval (*P< 0.05 vs control, Δ & +*P<*0.01 vs DSS-mice). (d) Damage score ranged from 0 to 4, scales judged based on the number and extent of PMN infiltration, epithelial defects, crypt loss, mucosal disruption, edema, and apoptosis, as described in the text

**Figure 7:**
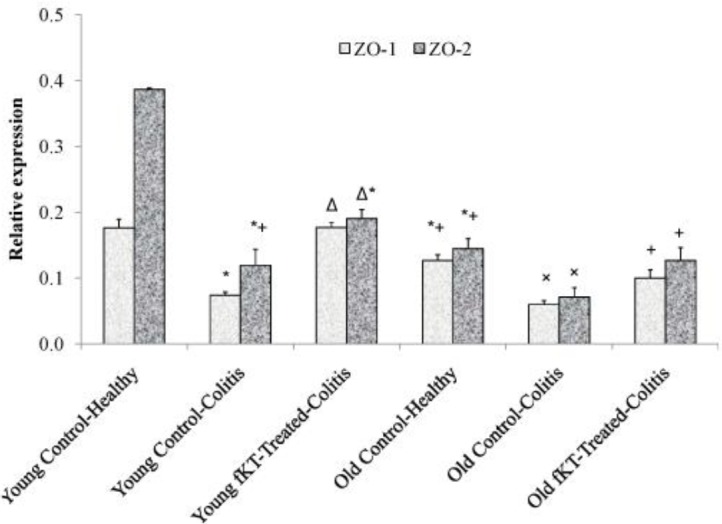
Comparison of ZO-1 and ZO-2 expression in the colons of young and old healthy, DSS-induced colitis, DSS-induced colitis treated with fKT. Treatment was performed on day 7 when the symptoms were observed and the disease flared up. Animals were sacrificed on day 14 and then RNA was extracted from the colon. The quantification of each gene was normalized against the reference gene GADPH. Oral administration of fKT assisted expression of ZO-1 and ZO-2 levels compared with the animals with colitis. Data are presented as mean±SD. (*): significant difference with young, healthy animals, (Δ): significant difference with young DSS-induced colitis, (+): significant difference with old DSS-induced colitis, (×): significant difference with old DSS-induced colitis treated with fKT (*P<*0.05)

**Figure 8:**
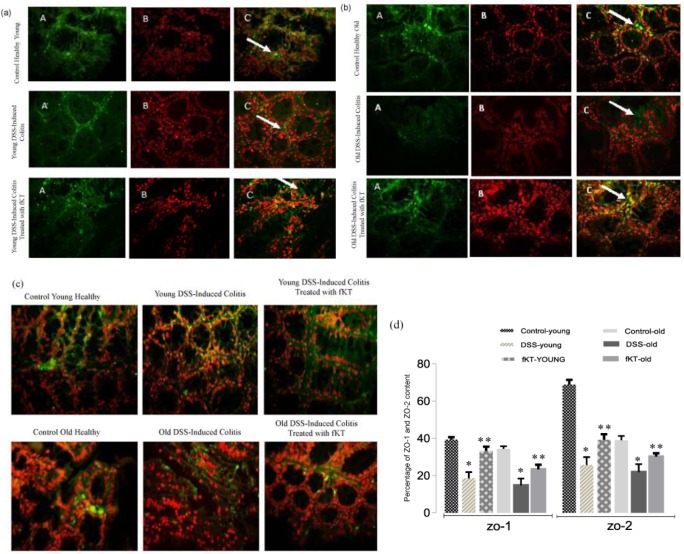
(a, b) Immune florescent-staining of ZO-1 protein expression (A: Primary antibody to ZO1, B: nuclei stain by PI, C: Merge A&B, Magnification: ×400) and (c) ZO-2 protein expression in the colons of mice. A positive reaction was observed as green staining. (d) The images were analyzed using image j scan software (imagej.nih.gov/ij, in 4 nonconsecutive tissue sections). Total expression of ZO-1 and ZO-2 was evaluated in the same fields. The ratio of the total expression of ZO-1 and ZO-2 to total tissue was evaluated and reported to percent. Data are presented as the mean of 5 randomly selected fields of microscopic view. (* *P<*0.07 vs control, ** *P<* 0.05 vs DSS-mice)

The animals were divided into two groups of young and old (Figure 1). Each group was then further subdivided into two groups (8 in each) including normal and colitis-induced. As the figure indicates, each of colitis-induced old or young groups was further subdivided into two subgroups, including colitis-induced with no treatment and colitis-induced treated with fKT. The study was performed in three phases (Figure 2). In the first step, DSS-induced colitis was set up in young (2 months) and old (16 months) mice in a period of 21 days during which weight loss and the clinical score were evaluated and compared with the age-matched healthy animals. In the second phase, the effect of fKT administration on survival analysis and the clinical score were evaluated in a period of 21 days. After completion of phase I and II of the study, molecular and histological evaluation were performed on young and old healthy controls, DSS-induced colitis, and DSS-induced colitis treated with fKT animals in Phase III. Considering the mortality rate and clinical signs that occurred in the animals with colitis, animals at this phase of the study were sacrificed on day 14 after the beginning of the study.

***Colitis induction***

Colitis was induced on day 0 using administration of drinking water containing 3.5% (w/v) dextran sodium sulfate salt (DSS) (40000 kDa, MP Biomedical, Eschwege, Germany) per mouse per day. The animals and clinical signs of disease were daily monitored, indicated by weight loss, occurrence of blood in the stool or around the rectum, and diarrhea until day seven after the colitis induction. Weight loss was determined by comparing the body weight for each mouse to the baseline body weight and expressed as a percentage of weight loss. Other symptoms were scored according to the previously suggested system by Siegmund *et al*. (25). Briefly, the different signs for stool consistency were scored as follows: score 0, well-formed pellets; score 2, pasty and semi-formed stools that did not adhere to the anus; score 4, liquid stools that did adhere to the anus. The different signs for bleeding were scored as follows: score 0, no blood measured using the Hemoccult system (Beckman Coulter); score 2, positive Hemoccult; score 4, gross bleeding. Animals with borderline scores were given a one-half score.

***Histological and histopathological analysis***

To perform histological evaluation of the colon, the animals were sacrificed under ether anesthesia after the last treatment with drinking water or fKT. Colon was initially flushed with 1x ice-cold phosphate buffered saline (PBS) to remove feces completely. Tissue samples of the colon were then removed, fixed in 10% buffered formalin, and processed for paraffin sectioning. Sections of about 5 μm thickness were taken and stained with Hematoxylin and Eosin (H&E). The stained sections were examined with an Olympus cX41 microscope and photographed using an Olympus D330 digital camera. Damage score ranged from 0 to 4 scale was judged based on: inflammation represented by number and extent of leukocyte infiltration, epithelial defects represented by the severity of damage to the epithelial cell layer, crypt atrophy estimated visually for the percent of atrophy in the crypts, edema, polymorphonuclear cells(PMNs) infiltration, and mucosal disruption (26).

***Immunofluorescence studies of ZO-1 and ZO-2 expression***

Sections of 5 µm paraffin-embedded colon tissues were prepared from each sample and then dewaxed, hydrated, and incubated in a protein block solution. Subsequently, the sections were incubated with the primary rabbit monoclonal ZO-1 or ZO-2 antibody (diluted 1:100 in 0.01 mol /L PBS; Zo-1: ab214228, Zo-2: ab2273, UK) followed by incubation with a goat anti-rabbit Alexa flour 488 (ab150077, Abcam, Cambridge shire, UK). The images were captured using a DeltaPix fluorescent microscope (Smorum, Denmark) and evaluated independently by two expert pathologists.

***Analysis of gene expression by real-time PCR***

Total RNA was extracted from ~50 mg of frozen colon tissue using guanidine/phenol solution (reagent lysis Qiazol-USA) according to the manufacturer’s instruction. The quality and quantity of RNA concentrations were monitored using a NanoDrop 2000c (Eppendorf, Germany). Then, 1 μg RNA was reversely transcribed to DNA using Thermo Scientific Revert Aid First Strand cDNA Synthesis Kit (Munich, Germany), according to the manufacturer’s instructions. The relative expression of mRNA for GAPDH, ZO-1, and ZO-2 was determined by preparing reaction mixer with PCR Master Mix (2X) (Amplicon-Denmark) and gene-specific primers with diluted cDNA and final volume made up to 10 μl with nuclease-free water. Quantification and analysis were carried out in ABI real-time PCR. The sequences of primers, designed by Integrated DNA Technologies, were forward 5′-TGTCCCACTTGAATCCCC-3′ and reverse 5′-TGTTTCCTCCATTGCTGTG-3′ for ZO-1 and forward 5′-CTCCCTCTTCACATCTGCTTC-3′ and reverse R: 5′-CTGTTACTTGCTTTGGTCTGG-3′ for ZO-2. The efficiencies for primers used in the study varied between 95% and 105%. Primer pairs were validated to ensure the correct size of the PCR product and the absence of primer dimers. The GAPDH gene was chosen as an internal control against which mRNA expression of the target gene was normalized (27). The resultant gene expression level was presented as 2^-ΔCt^, in which ΔCt was the difference between Ct values of the target gene and GAPDH (36). 

***Statistical analysis***

Statistical analysis was performed using Graph Pad Prism 7.01. Data are presented as means± SD. ANOVA was used to indicate any significant difference between the groups. Survival rates were illustrated using Kaplan–Meier plots and compared using the log-rank test. Value of *P* was considered statistically significant when it was less than 0.05.

## Results

***Characteristics and clinical course of DSS-induced colitis***

The DSS-induced colitis in mice is the common animal model to address the pathogenesis of colitis and to evaluate therapeutic approaches (28). This model was set up in our laboratory and monitored for a period of 21 days (Figure 2, Phase I). To do so, male NMR mice were administered 3.5% DSS in drinking water for seven days. The animals were checked daily during the period of the study for survival rate, weight loss, and clinical signs of colitis, including bleeding and diarrhea and compared with the age-matched healthy animals. Survival analysis of the young DSS-treated animals demonstrated that 66% and 33% of the animals were alive on days 7 and 14, respectively, and all were dead on day 21 (Figure 3a). In case of the old DSS-treated animals, survival analysis revealed that 66% and 50% of the animals were alive on days 7 and 14, respectively, and all were dead on day 21 (Figure 3b). There was a significant weight loss in the DSS-treated young and old groups compared to the age-matched healthy animals. The DSS-treated young mice had lost roughly 10% and 46% of their weight on days 7 and 14, respectively (Figures 4a, b). The DSS-treated old mice had lost roughly 13.5% and 15% of their weight on days 7 and 14, respectively (Figures 4c, d). As for digestive disorder signs, bleeding and diarrhea were observed in DSS-treated young and old mice on day 2 and 3 after DSS administration, respectively. These results demonstrate that DSS-treated young mice show more severe clinical signs and lower survival rate than the DSS-treated old mice.

***Treatment with fKT ameliorates DSS-induced colitis***

To evaluate the therapeutic effect of fKT on the leaky gut syndrome, young and old animals were randomly grouped, as shown in Figure 2, Phase II. The model was set up by administration of DSS for seven days, and then fKT was administered for the next 14 days. The animals were checked daily for survival rate, weight loss, and clinical signs of colitis, including bleeding and diarrhea and compared with the age-matched healthy animals. Survival analysis of the fKT-treated young animals with colitis demonstrated that 93% of the animals were alive on day 14 and only 33% were dead by day 21, comparing to the age-matched animals with colitis, which had all died at this time point of the study (Figure 3a). Treatment of old animals with colitis using fKT increased survival rate up to 83% compared to the age-matched animals with colitis of which 50% were alive on day 14 and all dead by day 21 (Figure 3b). In line with the increased survival rate, weight loss had decreased in young animals with colitis treated with fKT (Figures 4a, b). The fKT-treated young animals with colitis had lost roughly 10%, 15%, and 13.5% of their weight on days 7, 14, and 21, respectively. Weight loss became lower and significantly improved on day 14 comparing to the DSS-induced colitis young animals. The fKT-treated old mice had lost roughly 9.5%, 15%, and 15.8 of their weight on days 7, 14, and 21, respectively (Figures 4c, d). Digestive disorder signs, bleeding, and diarrhea were improved in fKT-treated young and old mice indicated by a significant decrease in the composite score of bleeding and diarrhea (*P*<0.05, Figures 5a, b). These results demonstrate that although young animals with colitis showed more severe clinical signs and less survival rate than the old mice with colitis, young mice with colitis responded better to fKT treatment than the old mice.

***Histological observations***

Histological analysis of H&E-stained tissue sections (Figure 6) of all DSS-challenged young and old mice showed increased infiltration of PMNs, cryptic loss, epithelial defect, mucosal disruption, apoptosis, edema, and mucosal thinness compared with the age-matched healthy mice. Treatment with fKT decreased the extent of damage, though it did not lead to a complete reversal to the healthy status by the regimen administered in this study. Notably, old healthy mice had more infiltration of PMNs, cryptic loss, and edema than the young, healthy animals. As depicted in Figure 6c, the mucosal thickness was decreased by DSS administration as the clinical score increased in both young and old with DSS-induced colitis.

***ZO-1 and ZO-2 mRNA and protein expression are elevated after fKT treatment***

ZO-1 is a peripheral membrane protein and has an important role in the formation of tight junction in between gut epithelial cells (29). Forming a complex with ZO-1 and ZO-2 is another molecule involved in tight junction formation between intestinal epithelial cells (30). To determine therapeutic potency of fKT, the animals were sacrificed on day 14, and ZO-1/ZO-2 level was evaluated. As Figure 7 shows, ZO-1 mRNA level was significantly up-regulated in colonic tissue of young and old animals treated with fKT comparing with the untreated age-matched animals with colitis (*P*<0.05). In addition, a significant difference in ZO-1mRNA level between young and old healthy animals was observed (*P*=0.04). Interestingly, treatment with fKT resulted in higher ZO-1mRNA level in the young animals rather than the old animals (*P*=0.05). Expression of ZO-2 mRNA was significantly decreased in the old and young DSS-induced colitis mice (*P*=0.05). Treatment with fKT markedly up-regulated ZO-2 mRNA expression in both young and old animals affected by colitis. Notably, ZO-2 levels in the young, healthy animals were markedly higher than those of the old healthy animals (*P*=0.004). It is noteworthy that the ratios of ZO-2/ZO-1 in young and old healthy animals were roughly 2 and 1, respectively (Figure 7). This result was confirmed by immunohistochemical analysis of ZO-1 and ZO-2 performed on tissue sections of colon isolated from healthy control mice and the animals affected by colitis without treatment and treated with fKT (Figure 8). 

## Discussion

DSS-induced colitis is a well-established experimental model with many similarities to human IBD. DSS acts like a detergent and disrupts the intestinal epithelial lining and loss of crypts, leading to alteration of epithelial structure and increased intestinal permeability. It follows the entry of luminal bacteria and associated antigens into the mucosa and infiltration of the inflammatory immune cells into the mucosal and submucosal areas. This leads to the dissemination of proinflammatory mediators into the underlying tissue and induction of intestinal inflammation, as shown by rises in the extent of diarrhea and rectal bleeding (28, 31). 

In clinical practice, several studies have documented that disruption of intercellular tight junctions known as the “leaky gut syndrome” can predict IBD, which includes Crohn’s disease (CD) and ulcerative colitis (UC) (16-18). From the molecular aspect, it has been proven that decreased expression of ZO-1 and ZO-2 play important roles in the formation and pathogenesis of the leaky gut syndrome (1, 32). Consequently, reduction of the increased intestinal permeability in leaky gut syndrome is an interesting target for improvement of the clinical status of IBD (17-18). According to our data, fKT effectively increased ZO-1 and ZO-2 levels in the colons of young and old DSS-induced colitis mice compared with the control mice with colitis. Notably, given the influence of age on the intestinal barrier function, it has been shown that ZO-1 and ZO-2 expression in the young, healthy control group was higher than that of the old healthy control group, which is consistent with the previous reports (14, 33). This is conceivable because microbiota changes with age, and these changes affect intestinal tight junction (6, 34). Nevertheless, there is a report indicating that age is not a relevant parameter to influence the intestinal barrier markers (35). However, in this study, it was found that ZO-1 and ZO-2 levels are decreased with age in healthy animals. This phenomenon is similar to the process that occurred in DSS-induced colitis. Such a decrease in ZO-1 and ZO-2 levels may explain the defect and lower degree of complexity in the intestinal tight junctions during aging and leaky gut phenomenon. Notably, the rate of decrement in ZO-2 in the young animals was more than that of the old animals after treatment with DSS. This is consistent with the ratios of ZO-2/ZO-1 in young and old healthy animals, which were roughly 2 and 1, respectively. This may explain the more severe clinical score in the young animals after treatment with DSS by decreased ZO proteins, especially ZO-2 content, compared with the old animals. In the current study, we also found that fKT could ameliorate the symptoms of DSS-induced colitis, including body weight loss, bleeding, diarrhea, and survival rate in young and old mice, which indicates the relative safety of fKT management. Although, DSS-treated young mice show more severe clinical signs and lower survival rate than the DSS-treated old mice, young mice with colitis responded better to fKT treatment than the old mice. Accordingly, histological examination demonstrated the beneficial effect of fKT for improvement of colitis. Neutrophil infiltration, as an indicator of inflammation (36), other pathologic appearances, such as epithelial defect, mucosal disruption, edema, and apoptosis were also decreased in fKT-treated mice with colitis. Histological comparison of the colon sections between old and young healthy animals revealed that cryptic loss and edema in the old healthy mice was more than the young. The striking point was that goblet cells in the old healthy mice were up to 50% more compared with the young, healthy animals. Intestinal epithelial cells may have been replaced by goblet cells, the consequence of which could be reduced intestinal absorbance by aging.

This study is the first one that reports the age-dependent difference in ZO-1 and ZO-2 content and performs a histological evaluation on the effect of fKT on an animal model of colitis. However, since “leaky gut syndrome” or intestinal permeability is a result of the inflammatory process, evaluation of immunologic parameters such as pro- and anti-inflammatory cytokines and receptors such as Toll-like receptors could better estimate therapeutic efficacy of candidate reagents. Further research is required to evaluate the effect of KT on such parameters in colitis and intestinal permeability.

## Conclusion

To date, no report has shown the effectiveness of fKT on young or old animal models of colitis. Our results reveal the promising medicinal value of KT and suggest it for the patients with long-lasting IBD, as these patients are susceptible to mucosal disruption, ulceration, and even colonic cancer.
